# SERS and Machine Learning-Enabled Liquid Biopsy: A Promising Tool for Early Detection and Recurrence Prediction in Acute Leukemia

**DOI:** 10.1021/acsomega.4c08499

**Published:** 2025-03-20

**Authors:** Fatih Oktem, Munevver Akdeniz, Zakarya Al-Shaebi, Gulsah Akyol, Muzaffer Keklik, Omer Aydin

**Affiliations:** † Department of Hematology, Faculty of Medicine, Erciyes University, 38039 Kayseri, Turkiye; ‡ Department of Biomedical Engineering, Erciyes University, 38039 Kayseri, Turkiye; § Nanothera Lab, Drug Application and Research Center (ERFARMA), Erciyes University, 38039 Kayseri, Turkiye; ∥ Clinical Engineering Research and Implementation Center (ERKAM), Erciyes University, 38040 Kayseri, Turkiye; ⊥ Nanotechnology Research and Application Center (ERNAM), Erciyes University, 38040 Kayseri, Turkiye

## Abstract

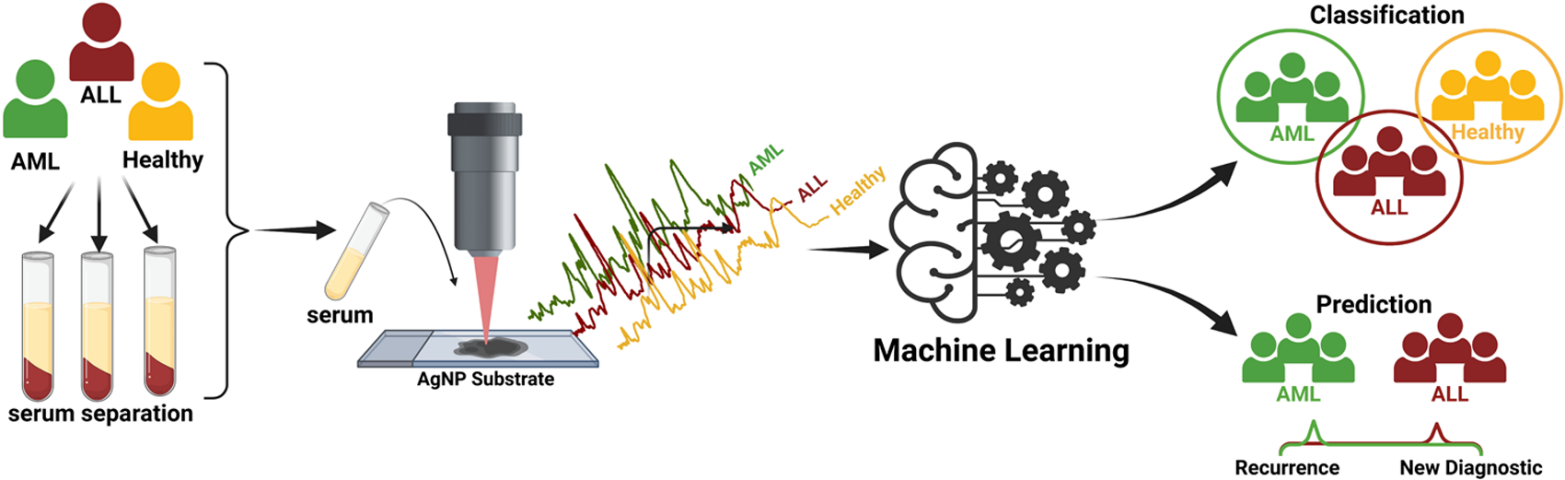

Acute leukemia (AL), classified as acute myeloid leukemia (AML) and acute lymphocytic leukemia (ALL), is a hematologic malignancy caused by the uncontrolled proliferation of leucocytes in the bone marrow. Early detection of AL is crucial for clinical treatment. Detection methods of AL are currently blood tests, bone marrow tests, imaging, and spinal fluid tests. However, these tests have drawbacks, such as high cost and time consumption. Liquid biopsy using biological fluids such as blood or serum is an emerging technique for noninvasive cancer detection and monitoring. Surface-enhanced Raman spectroscopy (SERS), which enhanced Raman signals by the interaction of plasmonic nanostructures with the analyte, is a highly sensitive and specific detection method with simple sample preparation that has been used in combination with machine learning techniques to analyze liquid biopsy. In this study, we developed a SERS-based liquid biopsy approach that enables accurate classification of AML and ALL subtypes and the prediction of disease recurrence. SERS spectra of serum samples from 24 healthy individuals, 43 AML patients, and 18 ALL patients were obtained using an Ag-based SERS substrate and clustered using hierarchical cluster analysis (HCA). The spectra were then classified using three commonly used classifiers, namely, support vector machine (SVM), random forest (RF), and k-nearest neighbor (kNN). Our findings demonstrate that the RF classifier has the highest accuracy values, with 96.1, 95.5, and 98.5% for classifying three groups and predicting the recurrence of AML and ALL, respectively. The combination of SERS-based serum analysis with machine learning algorithms represents a remarkable advancement in the realm of hematological disease diagnostics, particularly for AML and ALL. This approach not only facilitates the precise differentiation of disease subtypes but also introduces the novel capability of prognosticating disease recurrence.

## Introduction

Acute leukemia (AL) is a common type of cancer characterized by the rapid and uncontrolled growth of immature white blood cells, which interfere with the production of normal blood cells.^1^ There are two main types of acute leukemia: acute lymphoblastic leukemia (ALL) and acute myeloid leukemia (AML).^2,3^ ALL affects immature lymphoid cells, while AML results from an accumulation of immature myeloid blast cells in the bone marrow that often extends to the circulating blood.^4−6^ ALL is typical of pediatric age, while acute myeloid leukemia is more common in adult age. Early diagnosis of acute leukemia is critical for achieving successful treatment outcomes.

Acute leukemia, in terms of diagnosis, requires identification of the cell lineage involved in neoplastic proliferation and classification of leukemia cells based on the differentiation/maturation stages.^7,8^ For this purpose, complete blood count, peripheral smear, morphological evaluation from bone marrow aspiration material, cytogenetic/fluorescent *in situ* hybridization (FISH) examination, flow cytometry, polymerase chain reaction (PCR), and detailed examination of bone marrow aspiration and biopsy are required.^9,10^ However, these techniques have limitations, including the invasiveness of bone marrow biopsy, the need for skilled personnel, and the time-consuming nature of the analysis. For these reasons, it is desirable to develop new diagnostic tools that provide rapid, highly sensitive, and quantitative cell identification and differentiation from easily accessible body fluids.

Liquid biopsy is a new and more comprehensive method for obtaining reliable molecular information from disease. When cancer cells undergo apoptosis or necrosis, they release circulating tumor DNA (ctDNA) fragments into the blood or lymphatic circulation. In addition, they also release exosomes, which are small vesicles containing proteins and nucleic acids, into the circulation.^11,12^ This approach offers several advantages over traditional tissue biopsies, including the ability to perform serial sampling, the potential for real-time monitoring of disease progression and treatment response, and the possibility of detecting metastases at an earlier stage.^13^ CTCs, ctDNA or ctRNA, and exosomes are typically detected by liquid biopsy using polymerase chain reaction (PCR), next-generation sequencing (NGS), enzyme-linked immunosorbent assay (ELISA), fluorescence-activated cell sorting (FACS), and mass spectroscopy.^14,15^ However since these methods have disadvantages, such as requiring large samples and a costly and complex sample preparation process, fast and cost-effective methods are needed.

The combination of surface-enhanced Raman spectroscopy (SERS) and liquid biopsy has emerged as a promising noninvasive diagnostic tool for acute leukemia.^16,17^ SERS is an analytical technique that enhances Raman scattering due to the interaction of plasmonic nanoparticles and molecules.^18,19^ This allows for detecting and identifying small quantities of molecules, including cancer biomarkers, proteins, and nucleic acids.^20^ However, SERS poses a challenge in identifying molecular changes since even a slight variation in the spectrum can signify the emergence of molecular differences, making it quite difficult to discern them, especially in biological samples.^21^ By leveraging machine learning approaches, it becomes possible to overcome this challenge and classify different groups by analyzing large SERS data sets.^22^ This is achieved by identifying the minimum variation within the groups while simultaneously exploring the maximum variation between them.^23,24^ There are limited studies for investigating and detecting acute leukemia using SERS and liquid biopsy. For instance, Ye et al. reported the discrimination of AML subtype based on plasma using SERS.^25^ Han et al. investigated the serum of acute leukemia via SERS and principal component analysis (PCA).^26^ In another study, Duan et al. reported metabolic profiling of AML serum samples by combining Ag nanoparticle-based SERS with proton nuclear magnetic resonance (NMR) spectroscopy.^27^ In addition to acute leukemia, various types of cancers have been diagnosed using serum by SERS and machine learning analysis.^28−35^ For instance, Shao et al. used serum samples for the diagnosis of prostate cancer.^30^ Lin et al. investigated serum protein related to breast cancer by SERS and machine learning.^29^ Moisoiu et al. reported a diagnosis of kidney cancer from liquid biopsy.^34^ Furthermore, acute leukemia was investigated using cell, genomic DNA, cell lysate, blood, and bone marrow via SERS, Raman spectroscopy, and different machine learning algorithms.^28,30,31,36−49^ Based on these studies, SERS and liquid biopsy have the potential to detect and quantify biomolecules that are indicative of acute leukemia, such as specific genetic mutations or abnormal protein expression in blood or other bodily fluids. This noninvasive approach could potentially replace the need for bone marrow biopsies and provide early detection and diagnosis of acute leukemia, leading to improved treatment outcomes and patient care.

**Scheme 1:**
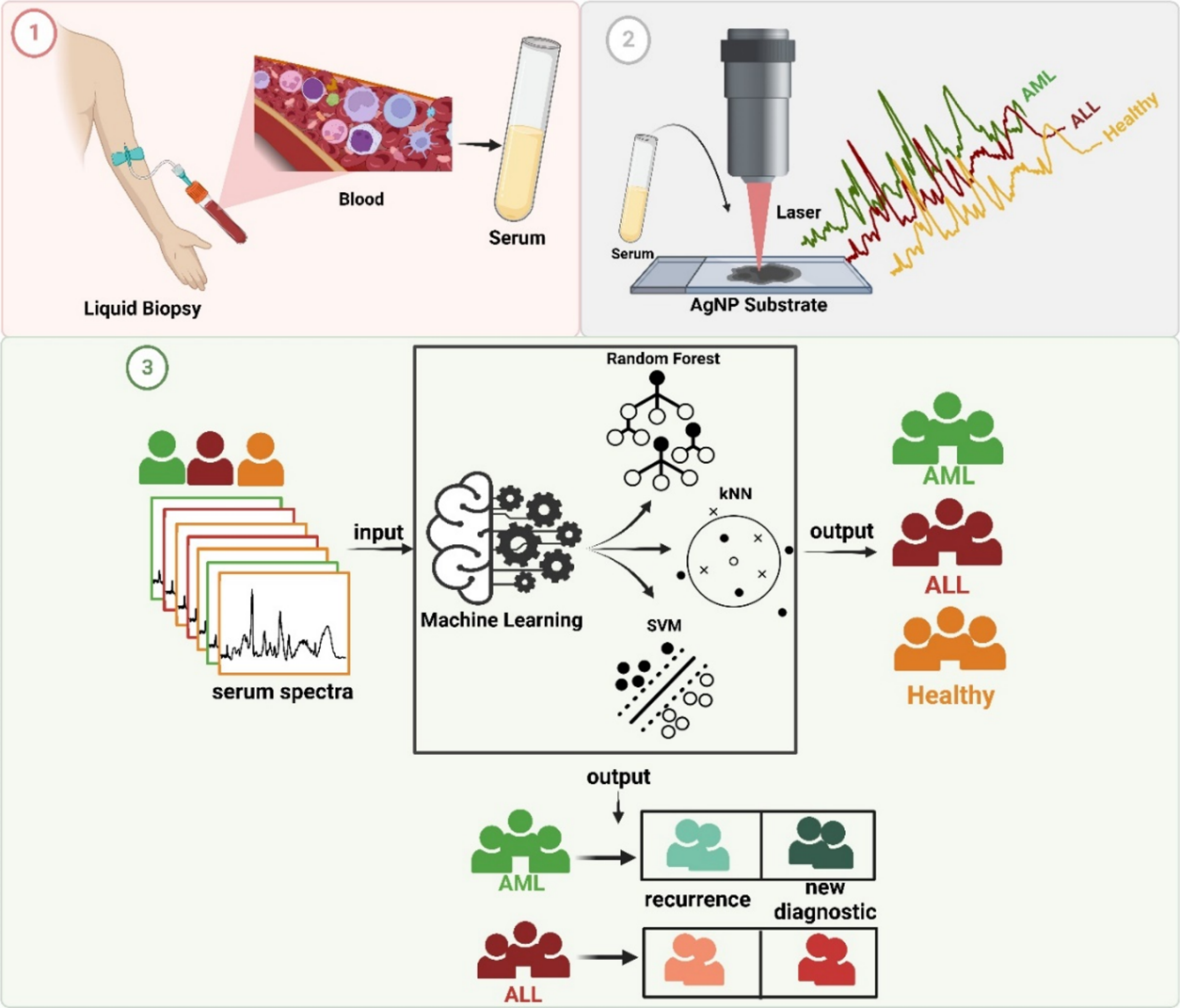
Schematic Illustration of General Workflow (1) collection of blood samples obtained from patients and healthy individuals, obtaining serum from blood, (2) collection of SERS spectra from serum samples using Ag-based SERS substrate, (3) machine learning models including RF, SVM, and kNN for classification of AML, ALL, and healthy serum, and prediction of recurrence of acute leukemia. (Created with BioRender.com.)

In this study, serum samples were obtained from patients diagnosed with AML and ALL, as well as from healthy individuals. SERS spectra were obtained from the serum samples using an Ag-based SERS substrate to generate hot spots. The SERS spectra were then analyzed by using machine learning with standard classifiers to differentiate between AML, ALL, and healthy samples. Furthermore, the machine learning algorithm was used to predict the recurrence of AML and ALL, as seen in Scheme 1. To the best of our knowledge, this study is the first to use SERS and machine learning approaches for both the classification of acute leukemia subtypes and the prediction of disease recurrence. These findings could have important implications for clinical trials and provide insights into the molecular differences underlying leukemia recurrence. The results may lead to a new clinical detection tool and help explain leukemia recurrence.

## Results and Discussion

### Identification of Acute Leukemia

**Figure 1:**
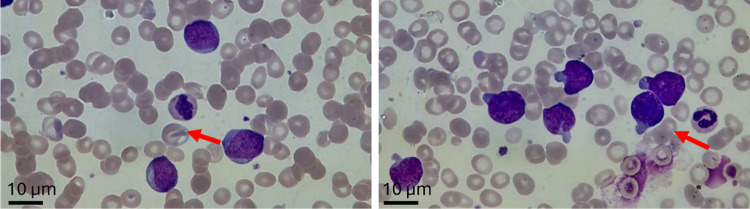
Blast cells in peripheral smears of AML and ALL patients, respectively. The red arrows indicate the presence of blast cells.

A total of 85 samples were classified into three groups for analysis, which included AML, ALL, and healthy control groups in this study. Diagnosis of patients was determined by peripheral smear, bone marrow aspiration, complete blood count, and immunophenotyping. The images of blast cells obtained from peripheral smears of patients with AML and ALL are shown with arrows in Figure 1. The percentages of blast cells in AML and ALL were determined by flow cytometry as shown in Figure S1. While CD117 and CD34 were selected markers of AML, CD19 and CD10 were selected markers of ALL.

Table 1 displays clinical features collected for each group, including routine laboratory test results and basic demographic information, such as age and gender. In addition, biochemical values such as total proteins, creatinine, and enzymes were also demonstrated. The Kruskal–Wallis test revealed significant differences (*p* < 0.05) in clinical features among the groups. The levels of hemoglobin, platelet, blood urea nitrogen, lactate dehydrogenase, total protein, prothrombin time, and International Normalized Ratio (INR) showed significant differences (*p* < 0.05) between the control group and the other groups. Among these variables, it was observed that hemoglobin and platelet values decreased approximately 1.5- and 3.5-fold in both AML and ALL groups compared to the control group, respectively. The levels of blood urea nitrogen were observed to increase by 1.5- and 2-fold in the AML and ALL groups, respectively. There was an increase in the level of lactate dehydrogenase (LDH) by 2.5- and 5-fold in AML and ALL groups, respectively, when compared to the control. Furthermore, the total protein values decreased by 1.2-fold in both AML and ALL groups. No significant difference was observed between ALL and AML for any of the remaining variables.

**Table 1:** Summary of the Routine Laboratory Test Results for Patients with AML, ALL, and Healthy Controls

	AML (*n* = 43)		ALL (*n* = 18)		control (*n* = 24)	
variable	value	range	value	range	value	range
age	56.60	23–82	39.91	18–64	31.79	24–45
gender	male: female	27–16	male: female	8–4	male: female	11–13
white blood cell (10^3^/μL)^a^	22.82	0.26–209.56	27.88	0.75–144.61	8.05	4.89–10.82
hemoglobin (g/dL)^a^	9.36	6.1–4.0	10.6	7.1–15.1	13.92	10–16.3
platelet (10^3^/μL)	76.28	13–699	136	25–477	293.63	202–475
blood urea nitrogen (mg/dL)^a^	17.01	3.7–58.5	24.89	3.9–104	11.18	6.40–16.3
creatinine (mg/dL)	0.90	0.34–2.16	1.18	0.57–6.04	0.75	0.51–1.20
lactate dehydrogenase (IU/L)^a^	548.28	103–3113	1000	155–5782	203.25	134–378
aspartate aminotransferase (IU/L)	26.42	8–117	43.8	10–191	19.21	10–34
alanine aminotransferase (IU/L)	25.44	4–87	31.66	8–82	18.75	8–49
total protein (g/dL)^a^	6.46	4.36–7.90	6.07	5–6.93	7.37	6.57–7.84
prothrombin time (s)^a^	12.44	10.3–18	11.61	9.8–16.4	11.36	10–13.4
International Normalized Ratio (INR)^a^	1.10	0.91–1.65	1.03	0.87–1.40	0.98	0.84–1.16
activated partial thromboplastin time (s)	25.40	16.2–49.3	26.15	20–41.7	26.78	21.9–30.6

a *p* < 0.05 between control and other groups. All *p*-values were corrected using the Bonferroni correction.

### Characterization of Silver Nanoparticles (AgNPs)

**Figure 2:**
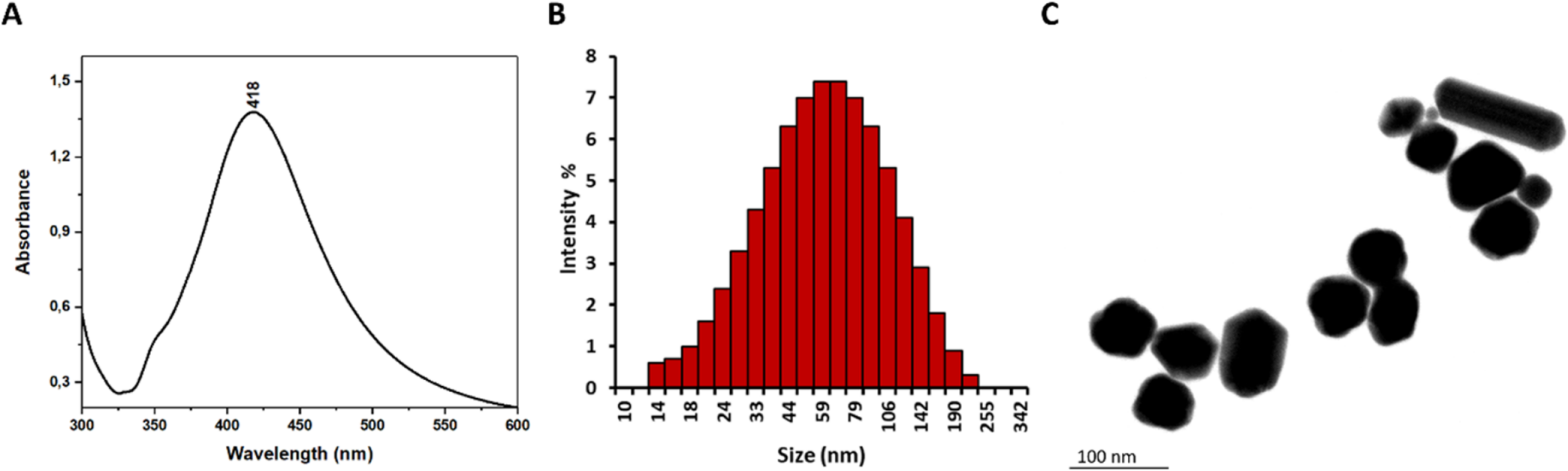
Characterization of AgNPs, (A) UV–vis spectroscopy, (B) DLS size distribution, and (C) STEM image.

To achieve SERS spectra that are both highly sensitive and reproducible, AgNPs were chosen as the material for the SERS substrate due to their capacity for exceptional optical enhancement and their simple preparation process.^50^ The synthesized AgNPs were characterized by utilizing a UV/vis spectrometer, dynamic light scattering (DLS), and STEM imaging. As can be seen in Figure 2A,B, the maximum absorption of AgNPs was measured at 418 nm, and the hydrodynamic size distribution of AgNPs was around 50–60 nm. The shapes of AgNPs were mostly spherical, as seen in Figure 2C.

Due to decreases in both the distance between nanoparticles and the interaction of samples with more nanoparticles, AgNPs were concentrated 16 times to form the SERS substrate.^51^ As shown in Figure S2, the distance between AgNPs was reduced with concentration. After 16× AgNPs were simply dropped on CaF_2_, the SERS substrate was ready for use in SERS measurements.

### SERS Measurement from Serum Samples

**Figure 3:**
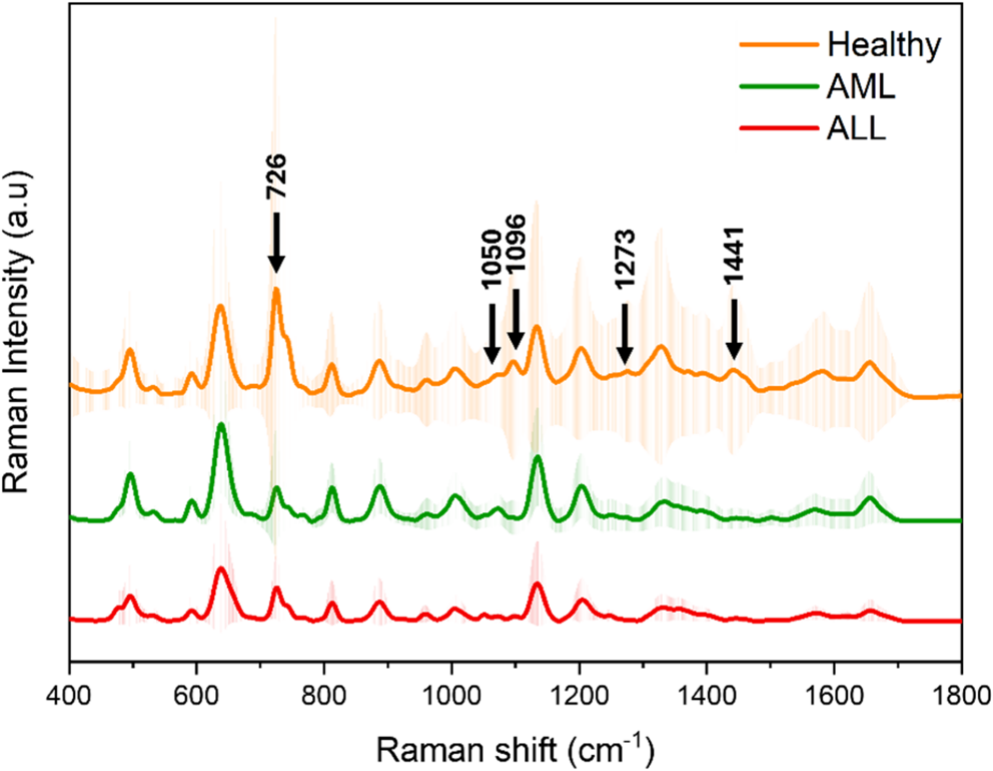
Comparison of mean AML, ALL, and healthy serum SERS spectra. SERS spectra underwent noise reduction via Cosmic ray removal, background subtraction (5th-degree polynomial), and Savitzky Golay smoothing (4th order, width 11 points)

Equal volumes of serum samples were deposited onto the SERS substrates from three different groups. A minimum of 100 spectra were obtained from each serum sample to identify the molecular differences in the spectra. The normalized mean SERS spectra of each group are illustrated in Figure 3. (The raw data of the SERS spectra of each group are shown in Figure S3.) Reproducibility of spectra is displayed by spot-to-spot and sample-to-sample measurements given in Figures S4–S5 for AML, ALL, and healthy serum spectra. The RSD coefficient was calculated according to the two peaks at 639 and 1135 cm^–1^ in all groups (Table S1). Thus, our results show reliable reproducibility (less than 20%) for obtained spectra.^52^ The peak assignments that contributed to the structural changes are demonstrated in Table 2. Çulha et al. investigated serum content regarding amino acids, proteins, and nucleic acids.^53^ They observed that spectral differences observed in the cancer group were correlated to the presence of cancer-associated biomarkers. This study demonstrated that the composition of serum varied according to the different types of acute leukemia. While the spectra of the serum samples showed comparable peaks, some peaks, such as those at 639, 726, 1050, 1096, 1273, and 1441 cm^–1^, exhibited altered intensities between the groups, along with slight differences in the spectral patterns. In the literature, the peak at 639 cm^–1^ has been reported to assign with tyrosine, thymine, and adenine, as shown in Table 2.^25,53^ Genetic mutations of Class III Receptor Tyrosine Kinases (RTKs), which have a significant impact on the prognosis of AML patients, prevent irregular proliferation and affect sensitivity of cells to apoptotic signals, lead to uncontrolled proliferation of undifferentiated myeloid cells.^54,55^ Mutations that occur may affect the adenine and thymine concentrations in the structure. The change in the peak intensity in the AML group may be due to genetic mutations in the RTKs.

**Table 2:** Peak Assignments of SERS Spectra For AML, ALL, and Healthy Serum, Compiled from Refs^25,53,60−63^^a^

Raman peaks (cm^–1^)	peak assignments
496	S–S stretching of l-Arginine
532	S–S stretching
590	ascorbic acid, amide VI
639	C–C twisting, tyrosine, thymine, adenine
726	C–H bending of adenine, coenzyme A, hypoxanthine
765	pyrimidine ring breathing of tryptophan
812	C–C–O stretching of phosphodiester bands in RNA, l-Serine
885	d-Galactosamine, glutathione
960	tyrosine
1004	C–C stretching of phenylalanine
1050	C–C of amino acids, C–O of carbohydrates
1073	C–N stretching (collagen)
1096	PO_2_ backbone, Phe
1135	C–N stretching of d-Mannose
1201	ring of tryptophan, phenylalanine
1254	adenine, amide III
1273	amide III
1328	C–H stretching of adenine
1369	tryptophan
1393	CH_3_ symmetric (lipid assignment)
1441	C–H deformation (protein and lipid)
1583	C=C bending mode of phenylalanine
1655	C–O stretching of amide I

a The table summarizes characteristic Raman shifts and their corresponding molecular vibrations as reported in the referenced studies, providing a comparative overview of spectral features associated with disease and healthy states.

The peak at 726 cm^–1^ has been reported to be assigned to adenine in the literature.^25,53^ This peak has the highest intensity in the healthy group; acute leukemia results in differentiation and uncontrolled proliferation of myeloid and lymphoblast cells in the bone marrow. In acute leukemias, especially in the AML group, the biochemical and cellular components of the bone marrow microenvironment also vary. Purine nucleotides and nucleosides are essential components of the bone marrow microenvironment of AML.^56^ Adenine nucleotides are released into the extracellular space from necrotic and inflammatory cells and cancer cells. Throughout neoplastic growth, adenine nucleotides and adenosine are abundant components of the tumor microenvironment. Based on this information, it is apparent that the peak density of adenine is greater in bone marrow that in serum. The observed reduction in peak density in the AML groups can be attributed to the high levels of adenine and its derivatives present in bone marrow. At the same time, energy metabolism in healthy and acute leukemia serum differs. It has been stated that the peak at 1050 cm^–1^ originates from C–C bonds in amino acids and C–O bonds in carbohydrates.^57^ The peaks in the AML and ALL groups are more intense than those in the healthy group. The peak intensity at 1096 cm^–1^ was higher in the healthy group among the groups. This peak is associated with PO_2_, that is, due to nucleic acids. The intensity of this peak can also be explained by mutations in RTK. The peak at 1441 cm^–1^ is due to the C–H bands in proteins and lipids, and the peak intensity is higher in the healthy group. Apart from the increased need for fatty acids for membrane synthesis, cell growth, and proliferation in cancer cells, AL cells, particularly AML, may undergo lipid catabolism.^58,59^

While the spectral differences in serum samples between the different disease and healthy states were evident, visual inspection alone was inadequate for distinguishing other significant compositional differences. Moreover, the visual interpretation of spectral differences may lead to misinterpretation. To address this, multivariate analysis techniques, including unsupervised and unsupervised methods, were employed to discriminate among the three different groups. Machine learning approaches, such as classification, clustering, regression, and dimension reduction, were used for this purpose.

### HCA Analysis of Serum Spectra

**Figure 4:**
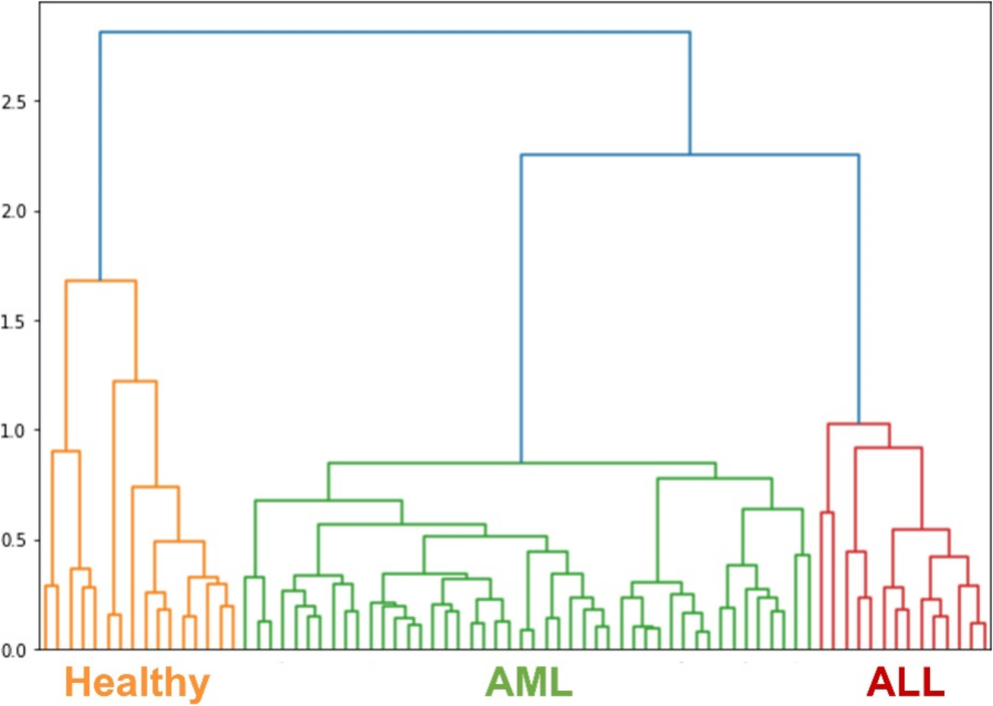
Dendrogram graph from HCA and SERS spectra shows the clustering of serum samples with different assigned groups. The batches of the different groups are marked in different colors.

In this study, the SERS spectra of serum samples from the different groups were clustered using HCA, which is an unsupervised technique that clusters data according to their distance or similarity. The clustering was performed by analyzing the content of biomolecules at different spectral positions in the serum SERS spectra, and the resulting dendrogram tree successfully clustered the serum spectra into three categories: AML, ALL, and healthy serums. The similarity of molecular structures in the serum samples was demonstrated by the distance and clustering, as illustrated in Figure 4.

HCA was performed by using Euclidean distance as the similarity metric. This method clustered the serum samples based on the intensity values of Raman peaks, which were subjected to preprocessing steps such as baseline correction, normalization, and smoothing. The Euclidean distance metric effectively distinguished between different spectral patterns, enabling us to group similar spectral profiles together and differentiate among AML, ALL, and healthy samples more clearly.

**Figure 5:**
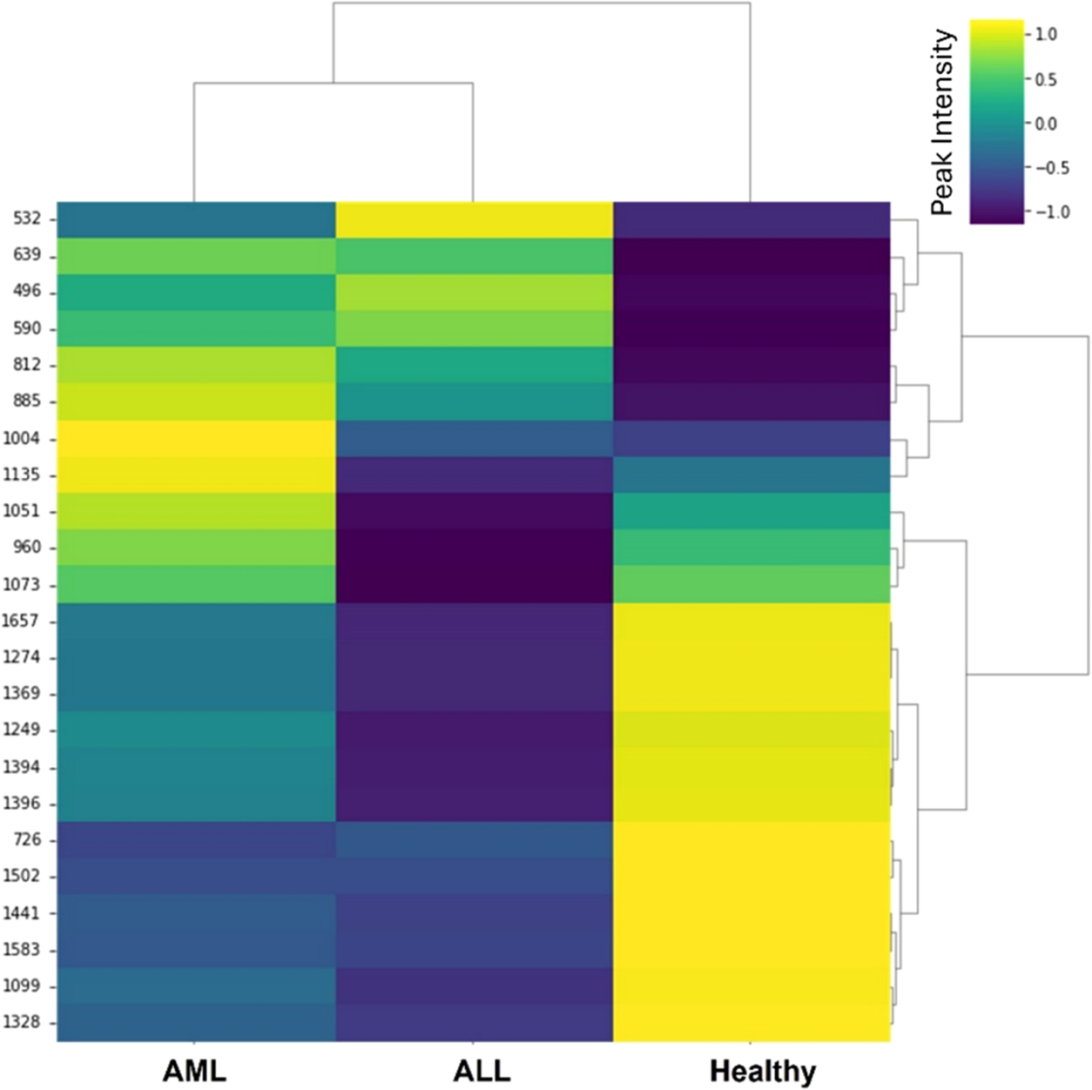
Heatmap visualization of the peaks of the serum spectra. The color bar in the upper-left corner shows the relative content gradient for the peaks.

The heatmap graph in Figure 5 demonstrates the intensity of peaks that varied between serum spectra. The graph was plotted based on peaks on the serum spectra, with yellow indicating peaks with the highest intensity and blue indicating peaks with the lowest intensity. According to the graph, the AML group had the highest density of peaks at 812, 885, 1004, 1051, and 960 cm^–1^, which could be used for AML detection. For the ALL group, the peaks with the highest intensity were at 496, 532, and 590 cm^–1^. In healthy serum spectra, the peaks at 726, 1099, 1249, 1274, 1328, 1369, 1394, 1396, 1441, 1502, 1583, and 1657 cm^–1^ had the highest intensity. The results obtained were consistent with the changes observed in the spectrum.

In general, HCA analysis allows the identification of similarities and differences among different groups of serum samples by clustering them based on their spectral characteristics. This analysis can reveal the presence or absence of specific Raman bands in each group and provide insights into the underlying molecular mechanisms that differentiate them. Moreover, the resulting dendrogram tree can help visualize the relationships between different groups of serum samples and identify potential outliers or subgroups.

### Machine Learning Analysis in AML, ALL, and Healthy Groups

The classification of AML, ALL, and health serum spectra is a crucial task in the diagnosis and treatment of blood-related diseases. In this study, we utilized three commonly used classifiers, SVM, RF, and kNN, to analyze and discriminate the unique spectral profiles of the three groups. Each of these classifiers has distinct advantages and disadvantages and can be used for both classification and regression tasks. SVM is a powerful model that can generate optimal hyperplanes or decision boundaries in a high-dimensional space, even in situations in which the boundaries are nonlinear and complex. The kernel trick used by SVMs allows them to define such complex boundaries by varying the type of kernel used, such as linear, polynomial, Gaussian, or RBF kernels, depending on the data set.^64^

On the other hand, kNN is a simple and easy-to-apply supervised learning algorithm that classifies based on the k value or similarity of the learning set and the nearest neighbor of the sample data point to be classified.^65^ Meanwhile, RF is a collective learning method for both classification and regression that classifies by generating multiple independent decision trees.^66^

The AML serum spectra data set consisted of 4300 spectra, the ALL data set included 1600 spectra, and the health data set comprised 2400 spectra. All of the spectra were taken from 85 samples of which 100 spectra were taken from an individual sample. 20% of the data, corresponding to 1600 spectra, was designated for testing the algorithms. To address class imbalance, we employed a random selection strategy to ensure an equal representation of each class in both training and testing data sets. Specifically, the data set was divided into 80% training and 20% testing subsets. During the random selection process, we ensured that each sample contributed a proportional number of spectra to the training and testing sets. This approach maintained the diversity of spectral variations within each class while avoiding overrepresentation of any particular sample. Additionally, we ensured that the random selection process maintained the natural distribution of spectral variations within each class. By performing multiple random splits and evaluating the class distributions, we confirmed that no class was disproportionately represented. This approach minimized potential biases and ensured a fair representation of all groups in both training and testing sets.

**Figure 6:**
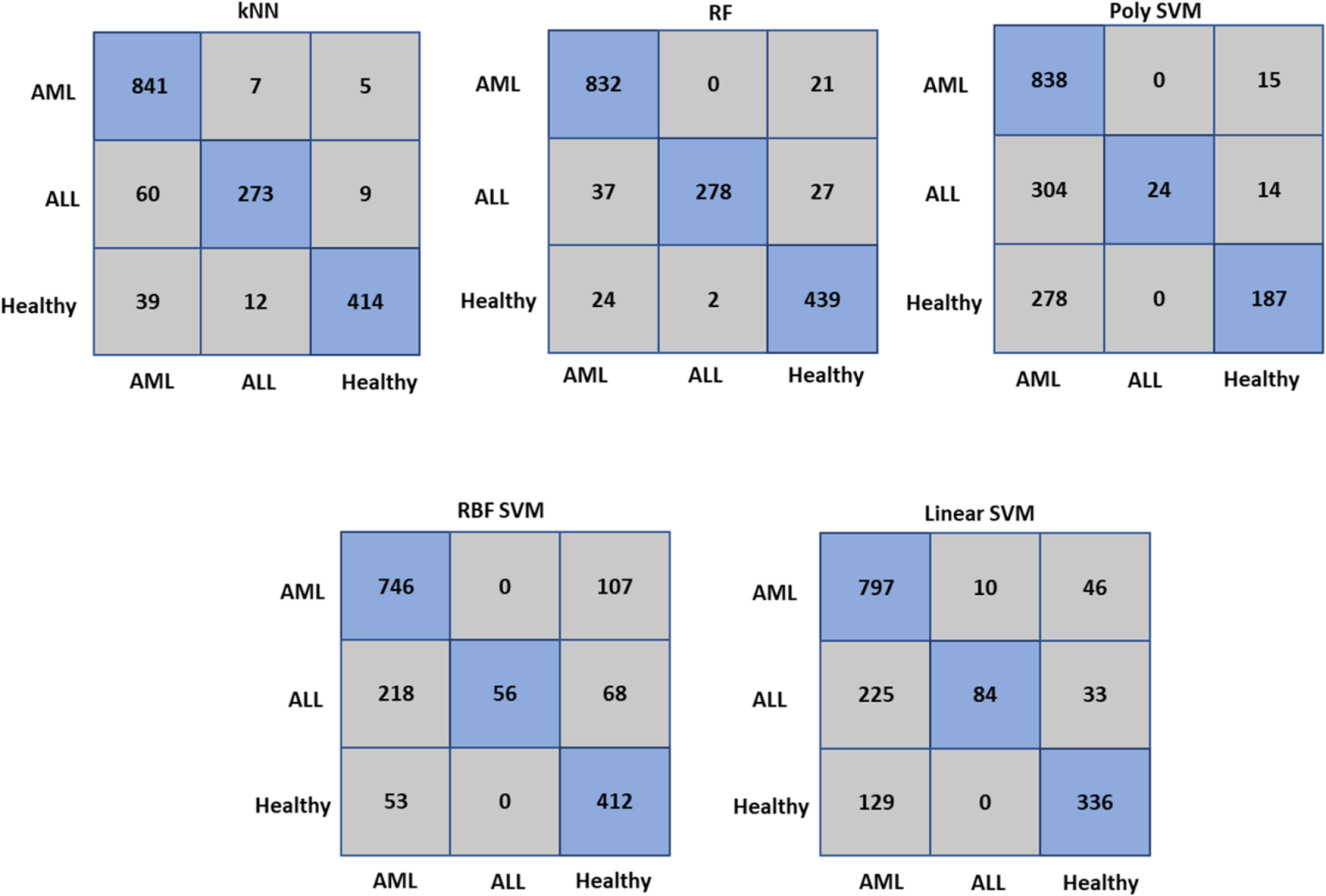
Confusion matrix of kNN, RF, poly SVM, RBF SVM, and linear SVM classifiers for classification of serum samples. Rows and columns of the matrix are actual and predicted classes, respectively. Diagonal elements illustrate classified serum spectra, while nondiagonal elements show misclassified serum spectra.

Our results showed that the RF classifier had the highest classification accuracy of 93.3%, while the lowest accuracy value was 63.2% in the Poly SVM and kNN classifiers for the three groups. The RBF SVM and linear SVM classifiers demonstrated accuracy values lower than those of RF, with accuracy values of 73.1 and 73.3%, respectively, as illustrated in Table 3. We calculated the accuracy values using a confusion matrix, which is a table used to describe the performance of a classification model by showing estimated values against actual values. The confusion matrix for each classifier is presented in Figure 6 for AML, ALL, and healthy serum spectra.

**Table 3:** Performance of the Classification Techniques for AML, ALL, and Healthy Serum Spectra Using the Test Data

		RF	kNN	linear SVM	RBF SVM	poly SVM
accuracy (%)		93.3	92.0	63.2	73.1	73.3
sensitivity (%)	AML	97.5	98.6	98.2	87.5	93.4
	ALL	81.3	79.8	7.0	16.4	24.6
	healthy	94.4	89.0	40.2	88.6	72.3
specificity (%)	AML	92.4	87.7	27.9	66.4	56.1
	ALL	99.9	98.6	100.0	100	99.2
	healthy	96.0	98.8	97.6	85.4	93.4
F1-score (%)	AML	95.3	93.8	73.7	79.8	79.5
	ALL	89.4	86.1	13.1	28.1	38.5
	healthy	92.2	92.7	54.9	78.3	76.4

The accuracy value was obtained with a confusion matrix. A confusion matrix is a table used to describe the performance of a classification model, showing estimated values against actual values. Each row of the confusion matrix represents actual values, and each column represents predicted values. Figure 6 demonstrates the confusion matrix of each classifier for AML, ALL, and healthy serum spectra.

According to the confusion matrix of the RF classifier with an accuracy of 93.3%, out of 853 AML spectra, 832 were correctly determined by the model, while 21 were classified as healthy spectra. 278 out of 342 of ALL spectra were correctly classified by the model, while out of the 465 healthy spectra, 439 were correctly classified. These results demonstrated the high performance of the RF classifier and its superiority over other traditional classifiers for the three groups.

### Investigation of Cancer Recurrence with SERS

**Figure 7:**
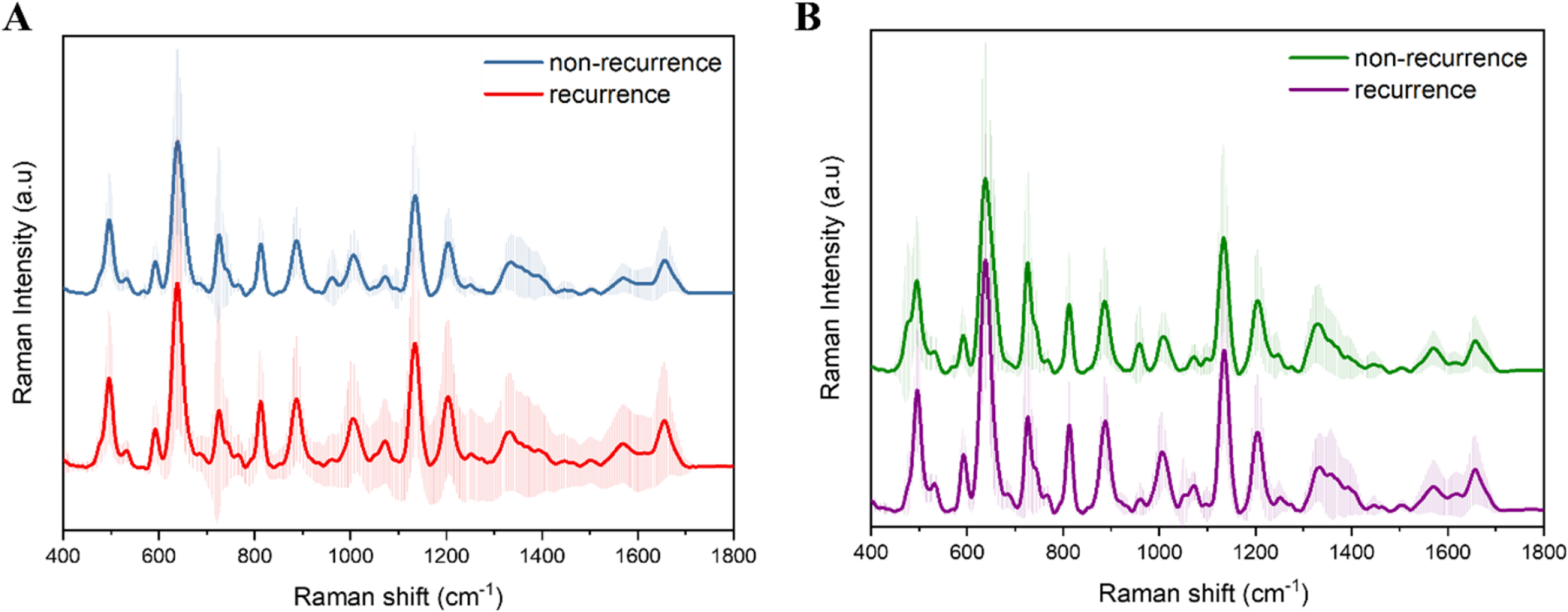
Comparison of serum spectra of newly diagnosed and recurrence patients in the (A) AML group and (B) ALL group.

If the cancer occurs after treatment, it is called a recurrence or recurrent cancer. Recurrent cancer begins with cancer cells that the initial treatment did not completely remove or destroy. This means that only a small number of cancer cells survive the treatment and are too small to show up in follow-up tests. Over time, these cells develop into tumors or cancers that are strong enough to detect them. In order to prevent recurrence, it is necessary to follow cancer after treatment continuously and develop methods that can detect up to a single cancer cell. Considering this information, a follow-up of recurrence from serum samples was investigated using SERS and machine learning methods. AML and ALL patients included newly diagnosed patients and recurrence patients. The differences in the spectrum of new diagnostic and recurrence leukemia patients may be related to the systemic chemotherapy given. For this reason, SERS spectra were obtained to determine spectral differences from serum samples of newly diagnosed and recurrence patients. For the AML cohort, we acquired 2100 spectra from newly diagnosed patients and 2200 spectra from recurrence cases. Similarly, within the context of ALL, we gathered around 800 spectra from newly diagnosed patients and 800 spectra from recurrence cases. This meticulous curation of data sets ensured a comprehensive representation of both new diagnostic and recurrence scenarios within the AML and ALL categories. Figure 7A displays the mean SERS spectra of newly diagnosed and recurrent patients belonging to the AML group. Although the obtained spectra generally have similar spectral profiles, some differences were observed in the serum samples where the disease relapsed, depending on the effect of the chemotherapy drug taken by the patients. Patients with relapsed disease in the AML group received cytarabine, which inhibits cell proliferation and stops the cell cycle, especially in the S phase, by affecting the DNA replication process. As depicted in Figure S6, the cytarabine (100 mg/mL) SERS spectrum did not appear to contribute to the serum spectrum. However, this drug, which acts on DNA, is expected to affect DNA-related Raman shifts in the serum spectra. In the spectra obtained from the AML group, the shift in 960 cm^–1^ was caused by tyrosine in the serum spectrum in which the disease relapsed is more intense than in the new diagnosis. The intensity of the Raman shift at 726 cm^–1^, which is associated with adenine, decreases compared with the newly diagnosed serum group. In contrast, the Raman shift at 639 cm^–1^, which is associated with tyrosine, is more intense in the new diagnosis spectrum. These spectral differences in Raman shifts may be due to the mechanism of action of chemotherapy drugs that patients receive.^67^

When the new diagnostic and recurrence serum spectra of the ALL group are examined, there are similar spectral profiles to those in AML (Figure 7B). However, the difference between these two spectra is more significant than in AML. The fact that they occur in different cells and that the chemotherapy drugs are different may explain these differences. The shift at 960 cm^–1^ caused by tyrosine was more intense in the newly diagnosed serum spectrum, while its intensity was decreased in the relapse serum spectrum. These results show that the chemotherapy drugs taken cause the densities of shifts.

Our analysis reveals that spectral differences observed between newly diagnosed and recurrent leukemia cases may primarily result from chemotherapy-induced biochemical changes rather than direct indicators of recurrence. While our SERS-based approach successfully differentiates these cases, further longitudinal studies are required to validate its predictive accuracy for true recurrence. Future research should focus on integrating additional clinical parameters and larger data sets to enhance the robustness of recurrence detection.

### Machine Learning Analysis in Newly Diagnosed and Recurrence

In addition to classifying the original serum samples, we also tested our machine-learning algorithms on newly diagnosed and recurrent ALL and AML serum samples. These samples were important to classify, as they represent different stages of diseases and can provide valuable information for treatment planning. To ensure the rigorous evaluation of our classifiers, we adopted a training and testing methodology. Specifically, 20% of the acquired spectra were set aside for testing, while the remaining 80% were utilized for training the machine learning models.

**Figure 8:**
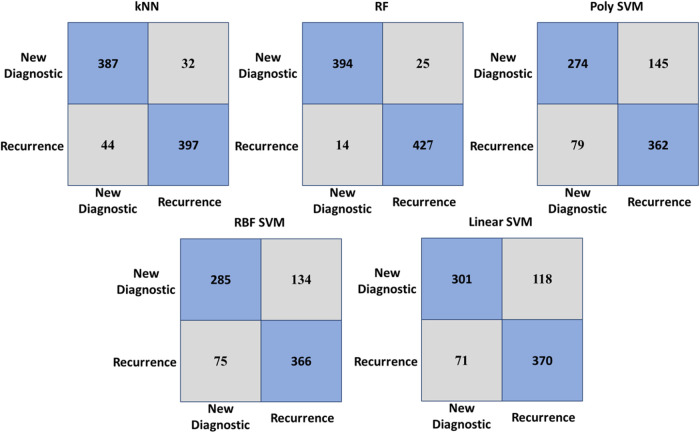
Confusion matrix of kNN, RF, poly SVM, RBF SVM, and linear SVM classifiers for classification of the newly diagnosed and recurrence AML serum spectra. Rows and columns of the matrix are actual and predicted classes, respectively. Diagonal elements illustrate classified serum spectra while nondiagonal elements show misclassified serum spectra.

The AML recurrence and newly diagnosed cases were also classified with high accuracy using the RF classifier with an accuracy of 95.5%. The other algorithms demonstrated lower accuracy values compared to the RF classifier, as shown in Figure 8 and Table 4.

**Table 4:** Performance of the Classification Techniques for the New Diagnostic and Recurrence of ALL and AML Serum Spectra Using the Test Data

	accuracy (%)		sensitivity (%)		specificity (%)		F1-score (%)	
	AML	ALL	AML	ALL	AML	ALL	AML	ALL
RF	95.5	99.7	96.8	100	94.0	99.3	94.9	99.7
kNN	91.2	95.7	90.0	92.3	92.4	99.3	91.3	95.7
poly SVM	74.0	98.4	82.1	98.8	65.4	98.0	76.4	98.5
RBF SVM	75.7	98.1	83.0	99.4	68.0	96.7	77.8	98.2
linear SVM	78.0	98.4	83.9	97.6	71.8	99.3	79.7	98.5

It is worth noting that for the KNN models presented in Tables 3 and 4, the value of k was set to 2, indicating that the two nearest neighbors were considered for classification. The training parameters used for the RF models included a maximum tree depth of 20 and 100 estimators. For the SVM models, we employed different kernels. The SVM with a polynomial kernel (degree 2) was trained for the binary classification task. Additionally, SVM models with a radial basis function (RBF) and linear kernel were utilized.

**Figure 9:**
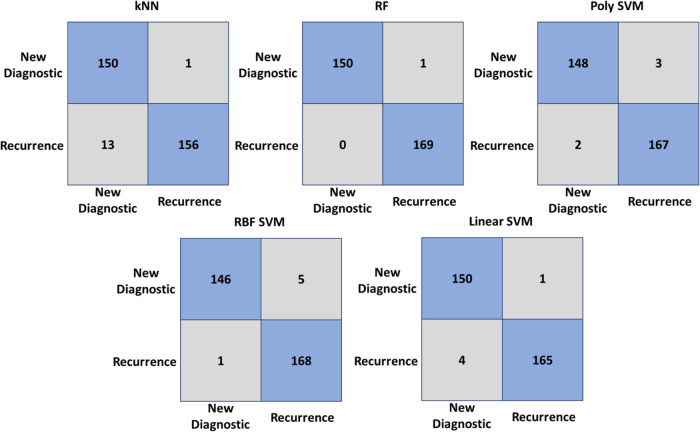
Confusion matrix of kNN, RF, poly SVM, RBF SVM, and linear SVM classifiers for classification of the newly diagnosed and recurrence ALL serum spectra. Rows and columns of the matrix are actual and predicted classes, respectively. Diagonal elements illustrate classified serum spectra while nondiagonal elements show misclassified serum spectra.

The confusion matrices of the ALL serum samples are shown in Figure 9, indicating the high accuracy achieved by the RF classifier with 99.7% accuracy. The kNN, poly SVM, RBF SVM, and linear SVM classifiers also performed well in this task, with accuracy values of 95.6, 98.4, 98.1, and 98.4%, respectively, as shown in Table 4.

Overall, our results suggest that the machine learning algorithms, particularly the RF classifier, can be used effectively to discriminate and classify newly diagnosed and recurrent cases of AML and ALL serum samples. These findings can have significant implications in the early diagnosis and treatment of blood-related diseases, potentially leading to improved patient outcomes.

In this study, it is important to address a notable limitation that could influence the interpretation and generalizability of our findings. The reduced number of available samples represents a constraint that may introduce biases and impact the validation process. While our methodology was meticulously designed to address this challenge, it remains a significant consideration.

The inherent limitation of a small sample size is its potential to yield results that could be influenced by random variation or sampling noise. Overfitting, a phenomenon where a model performs well on the training data but struggles to generalize to new data, becomes a concern. Additionally, the possibility of class imbalance could further exacerbate the limitations introduced by a restricted data set. To address these challenges, we implemented rigorous strategies such as the random selection and utilization of various machine learning algorithms.

## Conclusions

SERS and machine learning techniques provide insights into the underlying molecular changes in blood-related diseases such as AML and ALL. This study demonstrated that SERS combined with a machine-learning-based approach can detect acute leukemia and predict recurrence in AML and ALL. We collected serum samples from patients diagnosed with AML, ALL, and healthy individuals and analyzed them using an Ag-based SERS substrate. We employed three standard classifiers, SVM, RF, and kNN, to classify the SERS spectra and predict recurrence. Among them, the RF classifier demonstrated the highest accuracy values with 96.1, 95.5, and 98.5% for classification and recurrence prediction. The findings of this study suggest that SERS, combined with machine learning techniques, has the potential to be a reliable and accurate diagnostic tool for a range of blood-related diseases including AML and ALL. Future studies should investigate the feasibility of using this approach for clinical diagnosis and explore the possibility of expanding the range of diseases that can be diagnosed using this technique.

## Methods

### Materials

Silver nitrate (AgNO_3_, 99.0%) and sodium citrate (99%) were purchased from Sigma-Aldrich (UK). Calcium fluoride substrates (CaF_2_, 76 mm × 26 mm × 1.0 mm) were purchased from Crystran (Dorset, UK).

### Collection of Blood Samples and Preparation of Blood Serum

The Ethics Committee of the Erciyes University (Approval Date: 06/04/2022, Decision No: 2022/309) approved the study and written, and informed consent was obtained from all patients. Approximately 3 mL of human blood were collected from 24 healthy individuals as a control group, 43 AML patients, and 16 ALL patients without chronic disease. The control group, who was over 18 years old, did not have any chronic illnesses and did not use any medications for the 6 months prior to the blood sample collection. All blood samples were collected at the hematology department of Erciyes University Hospitals. To obtain blood serum, blood samples were centrifuged at 1900 rcf for 10 min at +4 °C. After centrifugation, blood serum was transferred to an Eppendorf tube and stored at −80 °C for the SERS measurement.

### Synthesis of Silver Nanoparticles and Preparation of the SERS Substrate

AgNPs were synthesized using the method reported by Lee and Meisel.^68^ Briefly, 90 mg of AgNO_3_ dissolved in 500 mL ultrapure dH_2_O was heated on magnetic stirrer until boiling. Ten mL 1% sodium citrate solution was added dropwise into the boiling solution and the solution was boiled for 1 h, followed by cooling to RT. The hydrodynamic diameter and ζ potential were measured using a Zeta-sizer (Malvern, Nano ZS, UK). Absorption spectra were obtained from AgNPs using a UV–visible spectrometer (PerkinElmer, Lambda 25) in the range of 300–700 nm. A nanoparticle tracking analyzer (NTA) instrument (Malvern, Nanosight NS300, UK) was used to determine the size distribution. Scanning transmission electron microscopy (STEM) images were acquired with a ZEISS GEMINI 500 instrument (Germany).

For fabrication of the SERS substrate, synthesized AgNPs were first centrifuged at 3200 rcf for 40 min. A portion of the supernatant was discarded to form 16× concentrated AgNPs (about 16 × 10^11^ particles/mL) to benefit from the advantage of the “coffee ring” effect which is aggregated metal nanoparticles in the ring region create many “hot spots”.^32,53^ Twenty μL of 16× AgNPs (about 3,2 × 10^10^ particles/mL–20 μL/cm^2^) were dropped onto CaF_2_ and dried at 23 °C for 2 h before SERS measurements. For each SERS measurement, the SERS substrate was prepared again.

### SERS Measurements

2 μL portion of the serum sample was dropped on the 20 μL/cm^2^ SERS substrate and dried at 23 °C for about 5 min. All SERS measurements were carried out using a WiTech α M+ Raman Microscopy System (WiTech α M+, Germany). The system was calibrated using a Si wafer, which has a Raman shift at 521 cm^–1^. Samples were excited with a near-infrared 785 nm diode laser with a laser power of 3 mW under a 50× objective lens (NA:0,8). Integration time was 5 s in all of the experiments. Each spectrum consists of 818 points from 400 to 1800 cm^–1^. Independent measurements were carried out for reproducibility under the same experiment conditions.

### Data Preprocessing and Machine Learning

The study involved obtaining 100 spectra from each serum sample from different points on the SERS substrate with mapping spectra. The resulting SERS spectra underwent noise reduction via cosmic ray removal, background subtraction (fifth-degree polynomial), and Savitzky Golay smoothing (fourth order, width 11 points) using WITec Project Plus 5.2 before normalization between 0 and 1. The data set was divided into two subsets: a training set and a test set. The training set comprised 80% of the total data set, and the remaining 20% constituted the test set. By using this 80:20 split, we aimed to assess the generalization performance of the machine learning models. Finally, machine learning analysis was performed on Google Colab using the Scikit-learn python library’s binary classifiers, including kNN, RF, and SVM with linear, radial basis function (RBF), and polynomial kernels, as well as HCA. The objective was to classify and cluster the spectra into AML, ALL, and healthy categories.

In selecting the machine learning algorithms, RF, SVM, and kNN, for our study, we aimed to leverage the unique strengths of each algorithm to address the complexities of SERS spectral data. RF was chosen for its robustness in handling noisy, high-dimensional data sets and its ability to provide feature-important scores for result interpretation. SVM, with its capacity to find optimal hyperplanes in high-dimensional space, was selected for its suitability for spectral analysis. Additionally, we included kNN to evaluate its performance in classifying SERS spectra and to make a comparative assessment with RF and SVM. These algorithm choices were based on their well-established effectiveness in similar applications and were intended to provide a comprehensive evaluation of machine learning techniques for leukemia classification and recurrence prediction using SERS data.

## Supporting Information Available

Experimental procedure including flow cytometry analysis and experimental results including blast cell distribution of AML and ALL patients by flow cytometry; STEM image of 16× AgNPs SERS substrate; raw data and preprocessing data of SERS spectra from AML, ALL, and healthy serum samples; relative standard deviation (RSD) of spot-to-spot and sample-to-sample measurements for two peaks; ten spot-to-spot SERS measurements of serum spectra; three sample-to-sample SERS measurements of serum spectra; cytarabine SERS spectrum (PDF)

## References

[ref1] Rose-Inman H.; Kuehl D. Acute Leuk. 2017, 31 (6), 1011–1028. 10.1016/j.hoc.2017.08.006. 29078921

[ref2] Balafar M. A.; Ramli A. R.; Saripan M. I.; Mashohor S. Review of brain MRI image segmentation methods. Artif. Intell. Rev. 2010, 33, 261–274. 10.1007/s10462-010-9155-0.

[ref3] Brunning R. D. Classification of acute leukemias. Semin. Diagn. Pathol. 2003, 20 (3), 142–153. 10.1016/S0740-2570(03)00031-5. 14552428

[ref4] Tallman M. S.; Gilliland D. G.; Rowe J. M. Drug therapy for acute myeloid leukemia. Blood 2005, 106 (4), 1154–1163. 10.1182/blood-2005-01-0178. 15870183

[ref5] De Kouchkovsky I.; Abdul-Hay M. Acute myeloid leukemia: a comprehensive review and 2016 update. Blood Cancer J. 2016, 6 (7), e441 10.1038/bcj.2016.50. 27367478 PMC5030376

[ref6] Terwilliger T.; Abdul-Hay M. Acute lymphoblastic leukemia: a comprehensive review and 2017 update. Blood Cancer J. 2017, 7 (6), e577 10.1038/bcj.2017.53. 28665419 PMC5520400

[ref7] Das P. K.; Jadoun P.; Meher S. Detection and Classification of Acute Lymphocytic Leukemia, In 2020 IEEE-HYDCON, Sept 11–12, 2020; pp 1–5.

[ref8] Raina R.; Gondhi N. K.; Chaahat; Singh D.; Kaur M.; Lee H.-N. A Systematic Review on Acute Leukemia Detection Using Deep Learning Techniques. Arch. Computat. Methods Eng. 2023, 30 (1), 251–270. 10.1007/s11831-022-09796-7.

[ref9] Haferlach T.; Kern W.; Schnittger S.; Schoch C. Modern diagnostics in acute leukemias. Crit. Rev. Oncol./Hematol. 2005, 56 (2), 223–234. 10.1016/j.critrevonc.2004.04.008. 16213152

[ref10] Haferlach T.; Bacher U.; Kern W.; Schnittger S.; Haferlach C. Diagnostic pathways in acute leukemias: a proposal for a multimodal approach. Ann. Hematol. 2007, 86 (5), 311–327. 10.1007/s00277-007-0253-2. 17375301

[ref11] Ignatiadis M.; Sledge G. W.; Jeffrey S. S. Liquid biopsy enters the clinic — implementation issues and future challenges. Nat. Rev. Clin. Oncol. 2021, 18 (5), 297–312. 10.1038/s41571-020-00457-x. 33473219

[ref12] Honoré N.; Galot R.; van Marcke C.; Limaye N.; Machiels J.-P. Liquid Biopsy to Detect Minimal Residual Disease: Methodology and Impact. Cancers 2021, 13, 5364 10.3390/cancers13215364. 34771526 PMC8582541

[ref13] Cheung A.-K.; Chow C.; To K. F. Latest development of liquid biopsy. J. Thorac. Dis. 2018, 10 (Suppl 14), S1645–S1651. 10.21037/jtd.2018.04.68. 30034830 PMC6035915

[ref14] Armakolas A.; Kotsari M.; Koskinas J. Liquid Biopsies, Novel Approaches and Future Directions. Cancers 2023, 15 (5), 1579 10.3390/cancers15051579. 36900369 PMC10000663

[ref15] Connal S.; Cameron J. M.; Sala A.; Brennan P. M.; Palmer D. S.; Palmer J. D.; Perlow H.; Baker M. J. Liquid biopsies: the future of cancer early detection. J. Transl. Med. 2023, 21 (1), 118 10.1186/s12967-023-03960-8. 36774504 PMC9922467

[ref16] Moisoiu V.; Iancu S. D.; Stefancu A.; Moisoiu T.; Pardini B.; Dragomir M. P.; Crisan N.; Avram L.; Crisan D.; Andras I.; Fodor D.; Leopold L. F.; Socaciu C.; Bálint Z.; Tomuleasa C.; Elec F.; Leopold N. SERS liquid biopsy: An emerging tool for medical diagnosis. Colloids Surf., B 2021, 208, 112064 10.1016/j.colsurfb.2021.112064. 34517219

[ref17] Zhang Y.; Mi X.; Tan X.; Xiang R. Recent Progress on Liquid Biopsy Analysis using Surface-Enhanced Raman Spectroscopy. Theranostics 2019, 9 (2), 491–525. 10.7150/thno.29875. 30809289 PMC6376192

[ref18] Alvarez-Puebla R. A.; Liz-Marzán L. M. SERS-Based Diagnosis and Biodetection. Small 2010, 6 (5), 604–610. 10.1002/smll.200901820. 20108237

[ref19] Sharma B.; Frontiera R. R.; Henry A.-I.; Ringe E.; Van Duyne R. P. SERS: Materials, applications, and the future. Mater. Today 2012, 15 (1), 16–25. 10.1016/S1369-7021(12)70017-2.

[ref20] Wang J.; Koo K. M.; Wang Y.; Trau M. Engineering State-of-the-Art Plasmonic Nanomaterials for SERS-Based Clinical Liquid Biopsy Applications. Adv. Sci. 2019, 6 (23), 1900730 10.1002/advs.201900730. PMC6891916 31832306

[ref21] Lussier F.; Thibault V.; Charron B.; Wallace G. Q.; Masson J.-F. Deep learning and artificial intelligence methods for Raman and surface-enhanced Raman scattering. TrAC, Trends Anal. Chem. 2020, 124, 115796 10.1016/j.trac.2019.115796.

[ref22] Al-Shaebi Z.; Akdeniz M.; Ahmed A. O.; Altunbek M.; Aydin O. Breakthrough Solution for Antimicrobial Resistance Detection: Surface-Enhanced Raman Spectroscopy-based on Artificial Intelligence. Adv. Mater. Interfaces 2023, 2300664 10.1002/admi.202300664.

[ref23] Ralbovsky N. M.; Lednev I. K. Towards development of a novel universal medical diagnostic method: Raman spectroscopy and machine learning. Chem. Soc. Rev. 2020, 49 (20), 7428–7453. 10.1039/D0CS01019G. 32996518

[ref24] dos Santos D. P.; Sena M. M.; Almeida M. R.; Mazali I. O.; Olivieri A. C.; Villa J. E. L. Unraveling surface-enhanced Raman spectroscopy results through chemometrics and machine learning: principles, progress, and trends. Anal. Bioanal. Chem. 2023, 415, 3945–3966. 10.1007/s00216-023-04620-y. 36864313 PMC9981450

[ref25] Ye M.; Chen Y.; Wang Y.; Xiao L.; Lin Q.; Lin H.; Duan Z.; Feng S.; Cao Y.; Zhang J.; Li J.; Hu J. Subtype discrimination of acute myeloid leukemia based on plasma SERS technique. Spectrochim. Acta, Part A 2022, 271, 120865 10.1016/j.saa.2022.120865. 35063821

[ref26] Han H.; Gong J.; Tian Y. Analysis of Serum from Acute Leukemia Patients Using Surface-Enhanced Raman Spectroscopy (SERS). Spectroscopy 2022, 37 (7), 36–41. 10.56530/spectroscopy.my4786e3.

[ref27] Duan Z.; Chen Y.; Ye M.; Xiao L.; Chen Y.; Cao Y.; Peng Y.; Zhang J.; Zhang Y.; Yang T.; Liu W.; Feng S.; Hu J. Differentiation and prognostic stratification of acute myeloid leukemia by serum-based spectroscopy coupling with metabolic fingerprints. FASEB J. 2022, 36 (7), e22416 10.1096/fj.202200487R. 35713583

[ref28] Feng S.; Wang W.; Tai I. T.; Chen G.; Chen R.; Zeng H. Label-free surface-enhanced Raman spectroscopy for detection of colorectal cancer and precursor lesions using blood plasma. Biomed. Opt. Express 2015, 6 (9), 3494–3502. 10.1364/BOE.6.003494. 26417518 PMC4574674

[ref29] Lin Y.; Gao J.; Tang S.; Zhao X.; Zheng M.; Gong W.; Xie S.; Gao S.; Yu Y.; Lin J. Label-free diagnosis of breast cancer based on serum protein purification assisted surface-enhanced Raman spectroscopy. Spectrochim. Acta, Part A 2021, 263, 120234 10.1016/j.saa.2021.120234. 34343842

[ref30] Shao X.; Pan J.; Wang Y.; Zhu Y.; Xu F.; Shangguan X.; Dong B.; Sha J.; Chen N.; Chen Z.; Wang T.; Liu S.; Xue W. Evaluation of expressed prostatic secretion and serum using surface-enhanced Raman spectroscopy for the noninvasive detection of prostate cancer, a preliminary study. Nanomed.: Nanotechnol., Biol. Med. 2017, 13 (3), 1051–1059. 10.1016/j.nano.2016.12.001. 27979746

[ref31] Yan B.; Li B.; Wen Z.; Luo X.; Xue L.; Li L. Label-free blood serum detection by using surface-enhanced Raman spectroscopy and support vector machine for the preoperative diagnosis of parotid gland tumors. BMC Cancer 2015, 15 (1), 650 10.1186/s12885-015-1653-7. 26438216 PMC4595250

[ref32] Hong Y.; Li Y.; Huang L.; He W.; Wang S.; Wang C.; Zhou G.; Chen Y.; Zhou X.; Huang Y.; Huang W.; Gong T.; Zhou Z. Label-free diagnosis for colorectal cancer through coffee ring-assisted surface-enhanced Raman spectroscopy on blood serum. J. Biophotonics 2020, 13 (4), e201960176 10.1002/jbio.201960176. 31909563

[ref33] Lin X.; Jia X.; Lin J. Y.; Wu P. H.; Weng Y.; Feng S. A comparative study based on serum SERS spectra in and on the coffee ring for high precision breast cancer detection. J. Raman Spectrosc. 2022, 53 (8), 1371–1379. 10.1002/jrs.6382.

[ref34] Moisoiu T.; Iancu S. D.; Burghelea D.; Dragomir M. P.; Iacob G.; Stefancu A.; Cozan R. G.; Antal O.; Bálint Z.; Muntean V.; Badea R. I.; Licarete E.; Leopold N.; Elec F. I. SERS Liquid Biopsy Profiling of Serum for the Diagnosis of Kidney Cancer. Biomedicines 2022, 10, 233 10.3390/biomedicines10020233. 35203443 PMC8869590

[ref35] Cai C.; Liu Y.; Li J.; Wang L.; Zhang K. Serum fingerprinting by slippery liquid-infused porous SERS for non-invasive lung cancer detection. Analyst 2022, 147 (20), 4426–4432. 10.1039/D2AN01325H. 36106390

[ref36] Managò S.; Mirabelli P.; Napolitano M.; Zito G.; De Luca A. C. Raman detection and identification of normal and leukemic hematopoietic cells. J. Biophotonics 2018, 11 (5), e201700265 10.1002/jbio.201700265. 29239116

[ref37] Zhao Y.; Zhang Y.; Liang X.; Liu S.; Cao X.; Chen N.; Chen Z.; Yan J. Monitoring the differentiation of dimethyl sulfoxide-induced human leukemia (HL-60) cells by Raman spectroscopy. J. Raman Spectrosc. 2021, 52 (6), 1086–1094. 10.1002/jrs.6122.

[ref38] Premasiri W. R.; Lee J. C.; Ziegler L. D. Surface-Enhanced Raman Scattering of Whole Human Blood, Blood Plasma, and Red Blood Cells: Cellular Processes and Bioanalytical Sensing. J. Phys. Chem. B 2012, 116 (31), 9376–9386. 10.1021/jp304932g. 22780445 PMC3704210

[ref39] Managò S.; Valente C.; Mirabelli P.; Circolo D.; Basile F.; Corda D.; De Luca A. C. A reliable Raman-spectroscopy-based approach for diagnosis, classification and follow-up of B-cell acute lymphoblastic leukemia. Sci. Rep. 2016, 6 (1), 24821 10.1038/srep24821. 27089853 PMC4835730

[ref40] González-Solís J. L.; Martínez-Espinosa J. C.; Salgado-Román J. M.; Palomares-Anda P. Monitoring of chemotherapy leukemia treatment using Raman spectroscopy and principal component analysis. Lasers Med. Sci. 2014, 29 (3), 1241–1249. 10.1007/s10103-013-1515-y. 24407964

[ref41] Moisoiu V.; Stefancu A.; Iancu S. D.; Moisoiu T.; Loga L.; Dican L.; Alecsa C. D.; Boros I.; Jurj A.; Dima D.; Bagacean C.; Tetean R.; Burzo E.; Tomuleasa C.; Elec F.; Leopold N. SERS assessment of the cancer-specific methylation pattern of genomic DNA: towards the detection of acute myeloid leukemia in patients undergoing hematopoietic stem cell transplantation. Anal. Bioanal. Chem. 2019, 411 (29), 7907–7913. 10.1007/s00216-019-02213-2. 31745615

[ref42] Hassoun M.; Rüger J.; Kirchberger-Tolstik T.; Schie I. W.; Henkel T.; Weber K.; Cialla-May D.; Krafft C.; Popp J. A droplet-based microfluidic chip as a platform for leukemia cell lysate identification using surface-enhanced Raman scattering. Anal. Bioanal. Chem. 2018, 410 (3), 999–1006. 10.1007/s00216-017-0609-y. 28905087

[ref43] Chen Y.; Jiang P.; Lei S.; Chen X.; Yao S.; Jiang D.; Lin D.; Jia X.; Hu J. Optical tweezers and Raman spectroscopy for single-cell classification of drug resistance in acute lymphoblastic leukemia. J. Biophotonics 2022, 15 (9), e202200117 10.1002/jbio.202200117. 35642096

[ref44] Leszczenko P.; Borek-Dorosz A.; Nowakowska A. M.; Adamczyk A.; Kashyrskaya S.; Jakubowska J.; Ząbczyńska M.; Pastorczak A.; Ostrowska K.; Baranska M.; Marzec K. M.; Majzner K. Towards Raman-Based Screening of Acute Lymphoblastic Leukemia-Type B (B-ALL) Subtypes. Cancers 2021, 13, 5483 10.3390/cancers13215483. 34771646 PMC8582787

[ref45] Chan J. W.; Taylor D. S.; Thompson D. L. The effect of cell fixation on the discrimination of normal and leukemia cells with laser tweezers Raman spectroscopy. Biopolymers 2009, 91 (2), 132–139. 10.1002/bip.21094. 18825777

[ref46] Jiang L.; Niu G.; Wu H.; Zhao J.; Liu Y.; Xie Z.; Yao Q.; Yu W.; Ren W.; Zhao G. Detection of K562 Leukemia Cells in Different States Using a Graphene-SERS Platform. ACS Appl. Nano Mater. 2021, 4 (9), 8972–8978. 10.1021/acsanm.1c01574.

[ref47] Ramoji A.; Neugebauer U.; Bocklitz T.; Foerster M.; Kiehntopf M.; Bauer M.; Popp J. Toward a Spectroscopic Hemogram: Raman Spectroscopic Differentiation of the Two Most Abundant Leukocytes from Peripheral Blood. Anal. Chem. 2012, 84 (12), 5335–5342. 10.1021/ac3007363. 22721427

[ref48] Paidi S. K.; Raj P.; Bordett R.; Zhang C.; Karandikar S. H.; Pandey R.; Barman I. Raman and quantitative phase imaging allow morpho-molecular recognition of malignancy and stages of B-cell acute lymphoblastic leukemia. Biosens. Bioelectron. 2021, 190, 113403 10.1016/j.bios.2021.113403. 34130086 PMC8492164

[ref49] Liang H.; Cheng X.; Dong S.; Wang H.; Liu E.; Ru Y.; Li Y.; Kong X.; Gao Y. Rapid and non-invasive discrimination of acute leukemia bone marrow supernatants by Raman spectroscopy and multivariate statistical analysis. J. Pharm. Biomed. Anal. 2022, 210, 114560 10.1016/j.jpba.2021.114560. 34999436

[ref50] Cialla D.; März A.; Böhme R.; Theil F.; Weber K.; Schmitt M.; Popp J. Surface-enhanced Raman spectroscopy (SERS): progress and trends. Anal. Bioanal. Chem. 2012, 403 (1), 27–54. 10.1007/s00216-011-5631-x. 22205182

[ref51] Kahraman M.; Yazici M. M.; Slahin F.; Bayrak Ö. F.; Çulha M. Reproducible Surface-Enhanced Raman Scattering Spectra of Bacteria on Aggregated Silver Nanoparticles. Appl. Spectrosc. 2007, 61 (5), 479–485. 10.1366/000370207780807731. 17555616

[ref52] Natan M.J. Concluding remarks surface enhanced Raman scattering. Faraday Discuss. 2006, 132, 321–328. 10.1039/b601494c. 16833126

[ref53] Avci E.; Yilmaz H.; Sahiner N.; Tuna B. G.; Cicekdal M. B.; Eser M.; Basak K.; Altıntoprak F.; Zengin I.; Dogan S.; Çulha M. Label-Free Surface Enhanced Raman Spectroscopy for Cancer Detection. Cancers 2022, 14, 5021 10.3390/cancers14205021. 36291805 PMC9600112

[ref54] K Bhanumathy K.; Balagopal A.; Vizeacoumar F. S.; Vizeacoumar F. J.; Freywald A.; Giambra V. Protein Tyrosine Kinases: Their Roles and Their Targeting in Leukemia. Cancers 2021, 13, 184 10.3390/cancers13020184. 33430292 PMC7825731

[ref55] Berenstein R. Class III Receptor Tyrosine Kinases in Acute Leukemia – Biological Functions and Modern Laboratory Analysis. Biomarker Insights 2015, 10s3, BMI.S22433 10.4137/BMI.S22433. PMC4527365 26309392

[ref56] Bakhtiyari M.; Liaghat M.; Aziziyan F.; Shapourian H.; Yahyazadeh S.; Alipour M.; Shahveh S.; Maleki-Sheikhabadi F.; Halimi H.; Forghaniesfidvajani R.; Zalpoor H.; Nabi-Afjadi M.; Pornour M. The role of bone marrow microenvironment (BMM) cells in acute myeloid leukemia (AML) progression: immune checkpoints, metabolic checkpoints, and signaling pathways. Cell Commun. Signaling 2023, 21 (1), 252 10.1186/s12964-023-01282-2. PMC10512514 37735675

[ref57] Ma W.; Lu J. An equivalence of fully connected layer and convolutional layer. 2017.

[ref58] Loew A.; Köhnke T.; Rehbeil E.; Pietzner A.; Weylandt K. H. A Role for Lipid Mediators in Acute Myeloid Leukemia. Int. J. Mol. Sci. 2019, 20 (10), 2425 10.3390/ijms20102425. 31100828 PMC6567850

[ref59] Stuani L.; Riols F.; Millard P.; Sabatier M.; Batut A.; Saland E.; Viars F.; Tonini L.; Zaghdoudi S.; Linares L. K.; Portais J.-C.; Sarry J.-E.; Bertrand-Michel J. Stable Isotope Labeling Highlights Enhanced Fatty Acid and Lipid Metabolism in Human Acute Myeloid Leukemia. Int. J. Mol. Sci. 2018, 19, 3325 10.3390/ijms19113325. 30366412 PMC6274868

[ref60] Xue L.; Yan B.; Li Y.; Tan Y.; Luo X.; Wang M. Surface-enhanced Raman spectroscopy of blood serum based on gold nanoparticles for tumor stages detection and histologic grades classification of oral squamous cell carcinoma. Int. J. Nanomed. 2018, 13, 4977–4986. 10.2147/IJN.S167996. PMC6124473 30214201

[ref61] Cao X.; Wang Z.; Bi L.; Zheng J. Label-Free Detection of Human Serum Using Surface-Enhanced Raman Spectroscopy Based on Highly Branched Gold Nanoparticle Substrates for Discrimination of Non-Small Cell Lung Cancer. J. Chem. 2018, 2018, 9012645 10.1155/2018/9012645.

[ref62] Gao N.; Wang Q.; Tang J.; Yao S.; Li H.; Yue X.; Fu J.; Zhong F.; Wang T.; Wang J. Non-invasive SERS serum detection technology combined with multivariate statistical algorithm for simultaneous screening of cervical cancer and breast cancer. Anal. Bioanal. Chem. 2021, 413 (19), 4775–4784. 10.1007/s00216-021-03431-3. 34128082

[ref63] Chen S.; Wang C.; Zhu R.; Zhu S.; Zhang G. Predicting prognosis in acute myeloid leukemia patients by surface-enhanced Raman spectroscopy. Nanomedicine 2021, 16 (21), 1873–1885. 10.2217/nnm-2021-0199. 34269596

[ref64] Xu Y.; Zomer S.; Brereton R. G. Support Vector Machines: A Recent Method for Classification in Chemometrics. Crit. Rev. Anal. Chem. 2006, 36 (3–4), 177–188. 10.1080/10408340600969486.

[ref65] Zhang S. Cost-sensitive KNN classification. Neurocomputing 2020, 391, 234–242. 10.1016/j.neucom.2018.11.101.

[ref66] Pan L.; Zhang P.; Daengngam C.; Peng S.; Chongcheawchamnan M. A review of artificial intelligence methods combined with Raman spectroscopy to identify the composition of substances. J. Raman Spectrosc. 2022, 53 (1), 6–19. 10.1002/jrs.6225.

[ref67] Niu Z.-X.; Wang Y.-T.; Sun J.-F.; Nie P.; Herdewijn P. Recent advance of clinically approved small-molecule drugs for the treatment of myeloid leukemia. Eur. J. Med. Chem. 2023, 261, 115827 10.1016/j.ejmech.2023.115827. 37757658

[ref68] Lee P.C.; Meisel D. Adsorption and surface-enhanced Raman of dyes on silver and gold sols. J. Phys. Chem. A 1982, 86 (17), 3391–3395. 10.1021/j100214a025.

