# Supplementary material for: SERS and Machine Learning-Enabled Liquid Biopsy: A Promising Tool for Early Detection and Recurrence Prediction in Acute Leukemia
Source: ACS Omega. 2025 Mar 20;10(12):11887–99. doi: 10.1021/acsomega.4c08499 (PMC11966330; doi:10.1021/acsomega.4c08499)
Supplement: Supplementary file 1 — ao4c08499_si_001.pdf [file ao4c08499_si_001.pdf]

Supplementary Information

SERS and Machine Learning-Enabled Liquid Biopsy: A Promising Tool for Early Detection and Recurrence Prediction in Acute Leukemia

Fatih Oktem [a,#], Munevver Akdeniz [b,c,#], Zakarya Al-Shaebi [b,c,#], Gulsah Akyol [a], Muzaffer Keklik [a], Omer Aydin,[b,c,d,e*]

a Department of Hematology, Faculty of Medicine, Erciyes University, 38039 Kayseri, Turkiye

b. Department of Biomedical Engineering, Erciyes University, 38039, Kayseri, Turkiye.

c. Nanothera Lab, Drug Application and Research Center (ERFARMA), Erciyes University, 38039 Kayseri, Turkiye.

d. Clinical Engineering Research and Implementation Center (ERKAM), Erciyes University, 38040, Kayseri, Turkiye.

e. Nanotechnology Research and Application Center (ERNAM), Erciyes University, 38040, Kayseri, Turkiye.

*** Corresponding Author**

Assoc. Prof. Omer Aydin

Department of Biomedical Engineering,

Erciyes University, 38039, Kayseri, Turkey

Phone: +90-352-207-6666 / Ext: 32984

E-mail: biomer@umich.edu; omeraydin@erciyes.edu.tr

#These authors contributed equally to this work.

Experimental

Flow Cytometry Analysis

Flow cytometry was performed by using FACS Calibur flow cytometer (Becton–Dickinson, Erembodegem, Belgium) by our previous published protocol[1]. Each related Abs was added to 100 µL of blood samples and incubated. While CD117 and CD34 markers were selected for AML, CD19 and CD10 markers were selected for ALL.

	Cell Distribution	%
AML	Neutrophil	10
	Monocyte	-
	Lymphocyte	10
	CD45 (Blast)	75
	CD45 Negative (Blast)	5
ALL	**Cell Distribution**	**%**
	Neutrophil	11
	Monocyte	0,1
	Lymphocyte	1
	CD45 (Blast)	88
	CD45 Negative (Blast)	-

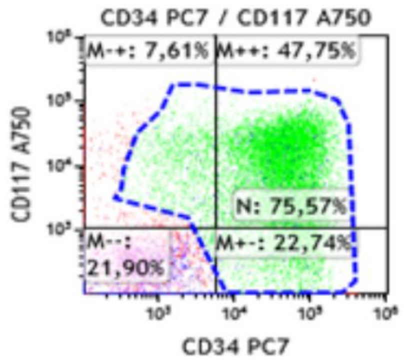

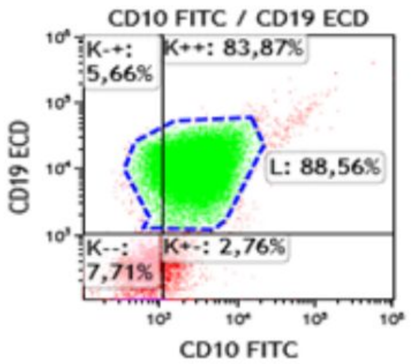

Figure S1. Blast cell distribution of AML and ALL patients by Flow cytometry. While CD117 and CD34 are marker of AML, CD19 and CD10 are marker of ALL.

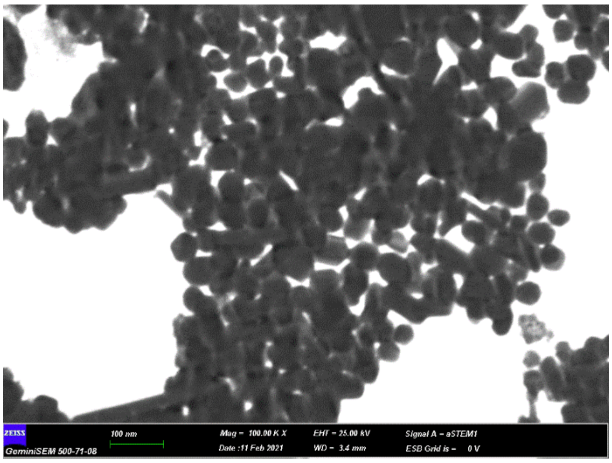

Figure S2. STEM image of 16x AgNP SERS substrate

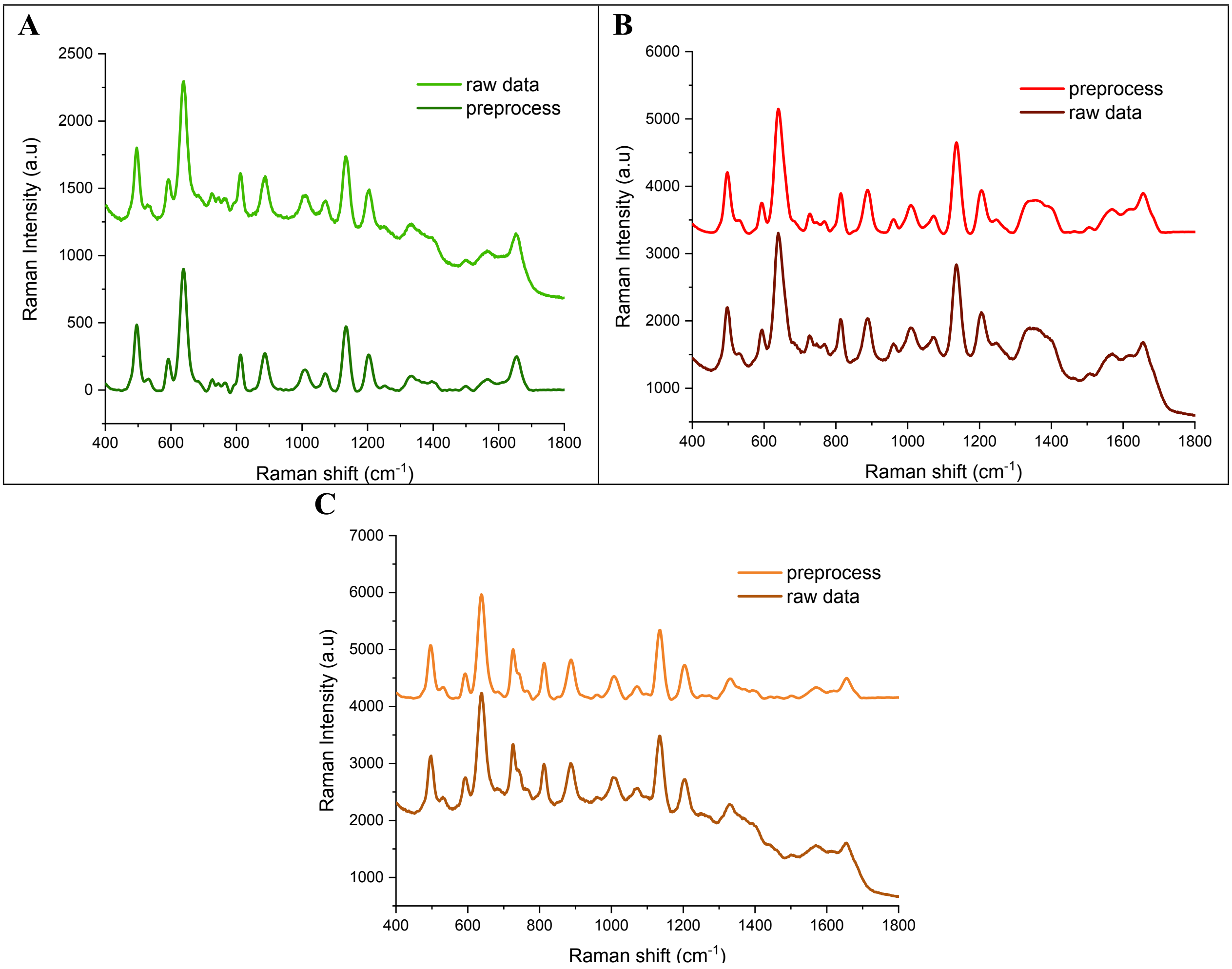

Figure S3. Raw data and preprocessing data of SERS spectra **A)** AML, **B)** ALL, and **C)** Healthy serum samples

Table S1. Relative standard deviation (RSD) of spot-to-spot and sample-to-sample measurements for two peaks (639, 1135 cm^{-1}) in AML, ALL, and healthy serum spectra.

**Spot-to-spot/Sample-to-sample**		**RSD (%) for 639 peak**	**RSD (%) for 1135 peak**
Spot-to-spot	**AML**	10.6	12.6
	**ALL**	10.4	10.7
	**Healthy**	4.9	4.0
Sample-to-sample	**AML**	10.4	10.9
	**ALL**	13.3	8.8
	**Healthy**	12.1	11.2

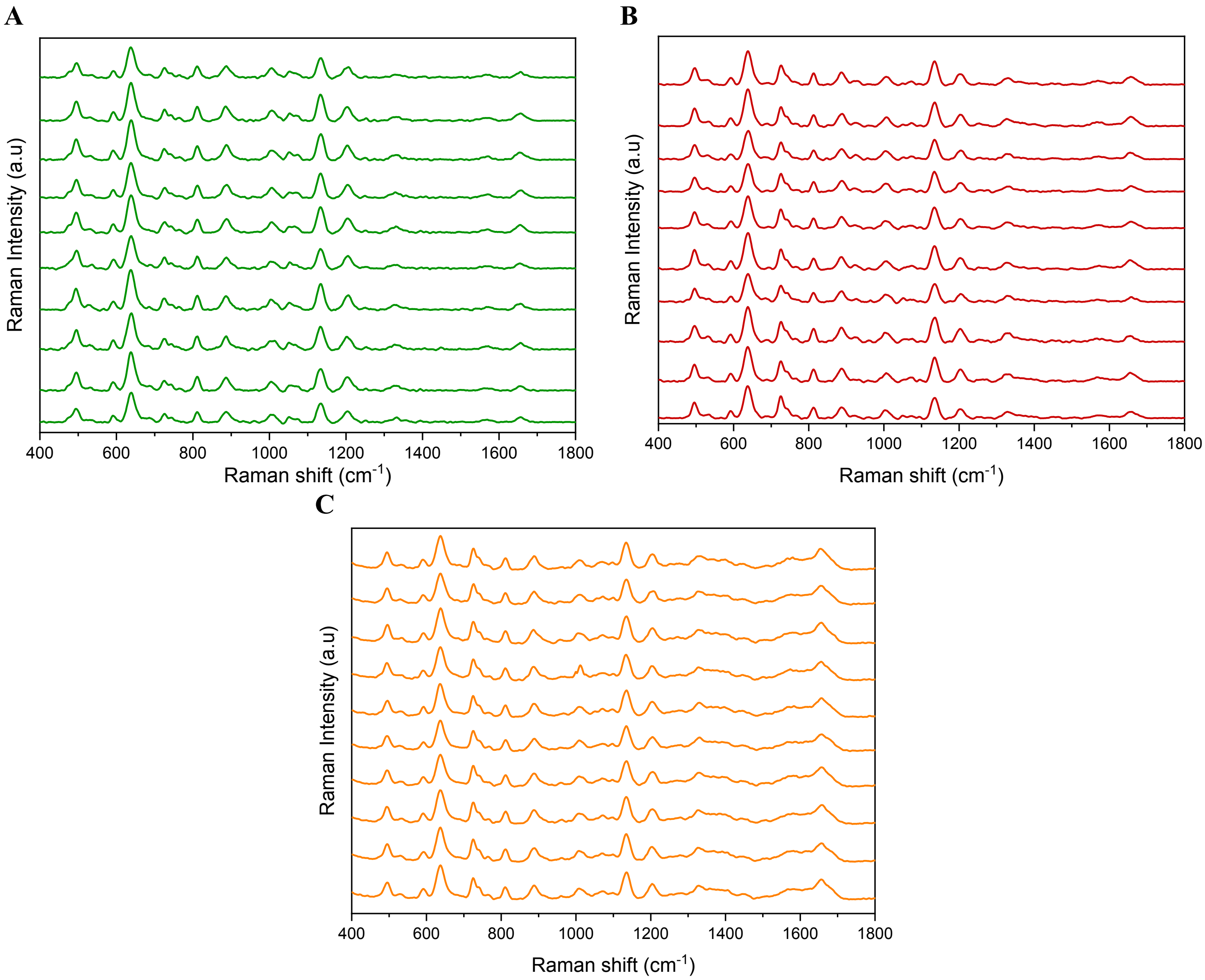

Figure S4. Ten spot-to-spot measurements of **A)** AML, **B)** ALL, and **C)** healthy serum spectra

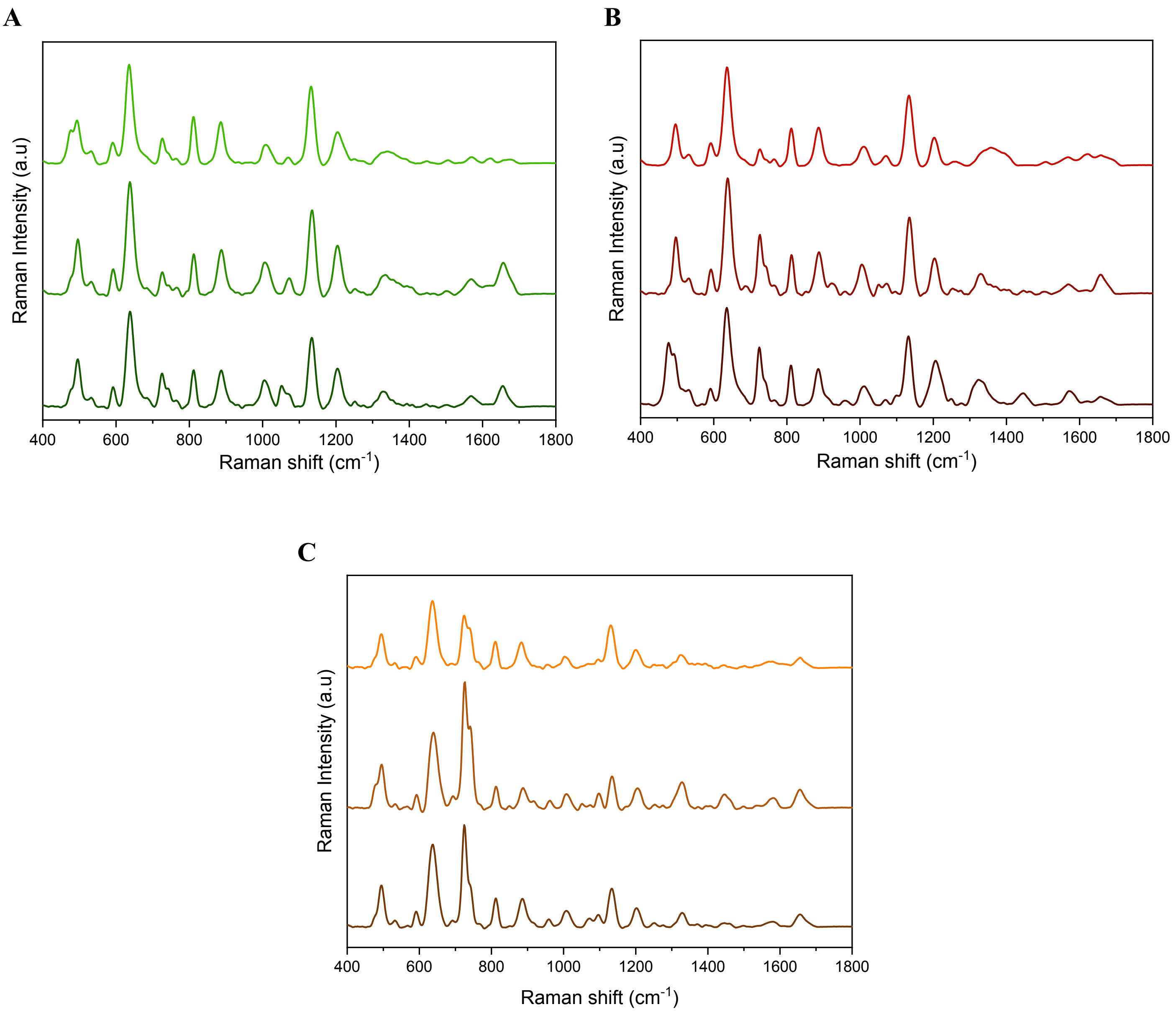

Figure S5. Three sample-to-sample measurements of **A)** AML, **B)** ALL, and **C)** healthy serum spectra.

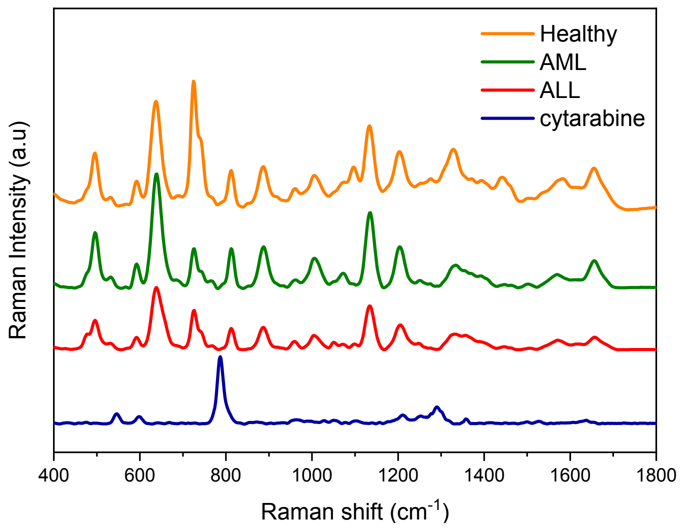

Figure S6. Cytarabine SERS spectrum

References

1. M. Keklik, S. Sivgin, B. S. Kalin, G. Akyol, C. Pala, M. Solmaz, L. Kaynar, B. Eser, M. Cetin and A. Unal, *Transfusion and Apheresis Science*, 2013, **48**, 293-295.
